# Absenteeism due to mental and behavioral disorder in employees of a federal university

**DOI:** 10.47626/1679-4435-2022-763

**Published:** 2023-02-13

**Authors:** Deisyane Fumian Bouzada, Núncio Antônio Araújo Sol, Camilo Adalton Mariano da Silva

**Affiliations:** 1 Programa de Pós-Graduação em Saúde e Nutrição, Universidade Federal de Ouro Preto, Ouro Preto, MG, Brazil

**Keywords:** mental disorders, occupational health, ML, universities, saúde do trabalhador, licença médica, transtornos mentais, universidades

## Abstract

**Introduction:**

Mental and behavioral disorders (MBD) are one of the main causes of absence from work in Brazil and worldwide.

**Objectives:**

To analyze the prevalence of absence from work according to the International Classification of Diseases, 10th revision, with stratification of disease defined as “Mental and Behavioral Disorders”, in permanent employees of the Federal University of Ouro Preto and its relationships with sociodemographic and occupational determinants, during the period from 2011 to 2019.

**Methods:**

An epidemiological, descriptive, and analytical study, with a cross-sectional design and quantitative approach was conducted using primary and secondary data. The population consisted of federal public sector workers who were granted ML to treat their own health during a 9-year period. Analyses were performed using descriptive and bivariate statistics. The Wilcoxon (Mann-Whitney) and Poisson tests were used to assess the existence of associations between variables.

**Results:**

733 medical records of employees eligible according to the inclusion criteria were analyzed. There was a rising trend in ML rates over the 9-year period. Of the sample, 23.2% (n = 170) were absent from work due to mental and behavioral disorders - females accounted for 57.6% and administrative technicians in education for 62.3%. In the multivariate analysis (Poisson test), only the outcome “time of first ML due to mental and behavioral disorders” was associated with the variable “time working at the Universidade Federal de Ouro Preto”.

**Conclusions:**

The high prevalence of mental and behavioral disorders found in this investigation is an alert to the magnitude of the problem, highlighting the urgency of implementing measures to detect psychosocial risk factors, whether associated with work or not.

## Abstract (pt)

**Introdução:**

Os transtornos mentais e comportamentais (TMC) configuram como uma das principais causas de afastamento do trabalho no Brasil e no mundo.

**Objetivos:**

Analisar a prevalência do afastamento do trabalho de acordo com a Classificação Internacional de Doenças e Problemas Relacionados à Saúde - 10ª Revisão, com estratificação da doença definida como “Transtornos Mentais e Comportamentais” dos servidores efetivos da Universidade Federal de Ouro Preto e sua relação com os determinantes sociodemográficos e ocupacionais, no período de 2011 a 2019.

**Métodos:**

Estudo epidemiológico com delineamento transversal e abordagem quantitativa, realizado a partir de dados primários e secundários. A população foi composta por servidores públicos federais com licenças médicas para tratamento da própria saúde no período de 9 anos. A análise foi realizada por meio de estatística descritiva e bivariada. Utilizou-se o teste de Wilcoxon (Mann-Whitney) e de Poisson para avaliação de existência de associação entre as variáveis.

**Resultados:**

Foram analisados 733 prontuários de servidores elegíveis nos critérios de admissão. A tendência das taxas de afastamento foi de crescimento no período de 9 anos. Na amostra, 23,2% (n = 170) apresentaram afastamento do trabalho por transtornos mentais e comportamentais - o sexo feminino apresentou 57,6%, e os técnicos administrativos em educação apresentaram 62,3%. Na análise multivariada (teste de Poisson), apenas o desfecho “momento do primeiro afastamento por transtornos mentais e comportamentais” apresentou associação com a variável tempo de Universidade Federal de Ouro Preto.

**Conclusões:**

A alta prevalência de transtornos mentais e comportamentais encontrada nesta investigação alerta para a magnitude do problema, evidenciando-se a urgência da implantação de medidas de detecção de fatores psicossociais de risco associados ou não ao trabalho.

## INTRODUCTION

According to the World Health Organization (WHO), mental health is a subjective state of wellbeing that enables people to realize their intellectual potential, establish satisfactory interpersonal relations, cope with crises in their lives, work productively, and contribute to their communities.^1^ Mental and behavioral disorders (MBD) are characterized by significant clinical and behavioral changes, by impaired functioning, or by any combination of these. They can cause clinically relevant suffering and harm to several areas of mental function and may derive from organic, social, genetic, chemical, or psychological factors.^2^

Working conditions have improved over recent years, as acquired labor rights have been delivered, combined with transformations that are taking place in the work scenario. It can be observed that greater care is being taken with cleanliness, health standards, and prevention of accidents. However, new forms of sickness are being observed, linked to workers’ psychological functioning and constituting a new and complex challenge for occupational health services. Effectively, the world of work is becoming ever more complex and multifaceted.^3^ The elevated frequency and prevalence of MBD in workers from many different occupational categories in Brazil and worldwide makes them one of the primary causes of days absent from work, with repercussions for individuals and for society.^4^ Classifying ML by economic sector and International Classification of Diseases and Problems Related to Health, 10th revision (ICD-10) categories, in the public sector in general, in 2018 MBD were the second-ranked cause of sickness benefit or ill-health retirement (16%) and the third-ranked cause of accident benefits (23%).^5^ Research data from high, middle, and low income countries estimate the annual prevalence of common mental disorders in the working population at 17.6%.^6^ In the European Union, it is estimated that 164.8 million people of all ages (38.2% of the population) suffer from MBD every year. The most common MBD are anxiety (14%), followed by insomnia (7%) and depression (6.9%), with a high financial cost due to lost productivity, absenteeism, or other costs.^6^ In 2016, in the United Kingdom, MBD were responsible for 15.8 million work days lost, or 11.5% of all work absenteeism due to disease in the country.^6^ Sickness due to MBD, which causes a high proportion of ML granted for health treatment, causes significant negative personal, institutional, economic, and social repercussions, such as work absenteeism, reduced capacity to work, and lost productivity.^7^ There are few scientific papers published on absence from work, and particularly on absenteeism due to MBD in public sector workers at educational institutions, although some advances have been made. In view of the impact of MBD and their repercussions for community health, studies on the subject are needed to support debate about the conditions of health and disease among workers.

The objective of this study was to analyze the prevalence of absenteeism from work, according to the ICD-10, with stratification of diseases defined as Mental and Behavioral Disorders (ICD-10, Chapter V), in permanent workers at the Universidade Federal de Ouro Preto (UFOP) and its relationships with socio-organizational and epidemiological determinants, during the period from 2011 to 2019.

## METHODS

### STUDY POPULATION

The study population comprised federal public sector workers (faculty members and educational technical and administrative workers [ETAW]) from the permanent staff of UFOP who were granted medical leave (ML) to treat their own health from 2011 to 2019. ML is a statutory provision that is granted to a worker who is unable to perform the tasks inherent to their job because of health reasons.

### STUDY DESIGN

This is a descriptive and analytical epidemiological study with a cross-sectional design and a quantitative approach, conducted using primary and secondary data.

### STATISTICS

Two databases were compiled with primary and secondary data. The database containing the primary information was populated with variables of interest (socio-organizational and epidemiological) collected from the medical records of workers who had time off from work from 2011 to 2019 for conditions in any of the groups in the ICD-10. These data were then screened to include only absences for ML listed in Chapter V of the ICD-10 - Group F (MBD), which constituted the database containing the variables of interest (sex, age group, occupational category, induction date, date of birth, number of absences for MBD, date of absence for MBD, number of days of absence and medical diagnosis). The personal history of absences and information on the variables chosen were extracted from medical records. Organizational data (date of induction to the UFOP staff and occupational category) were extracted from the Federal Government Transparency Portal, accessing the public domain page on civil and military staff of the Federal Executive Power. Data were input to spreadsheets, stored electronically (Excel, Microsoft Office 2019), and analyzed with Stata, version 14.0. A descriptive analysis of numerical variables was conducted, with absolute frequencies, percentages, means, with standard deviations (SD), and medians with maximum and minimum values. Nominal variables were expressed as absolute numbers and proportions. An exploratory univariate analysis identified explanatory variables of interest (sex, occupational category, age group, and time working at UFOP) that were significantly associated with each of the outcome variables (“number of absences for MBD”, “date of first absence for MBD”, and “total number of days absent for MBD”). Associations between these outcomes and each of the explanatory variables were identified using the Wilcoxon test (Mann-Whitney) to compare medians when the independent variable was dichotomized and the Kruskal-Wallis test when more than two groups were being compared.^8^ Variables that exhibited significant statistical associations in the univariate analysis (p < 0.20) and certain variables that the literature considers important in association with the events in question were selected for multivariate Poisson regression (prevalence ratio) to test their independent effects on the three dependent study variables. After selection of the independent variables in univariate analyses, a complete model was constructed for each of the three dependent variables, incorporating all of the explanatory variables selected earlier, which were then successively removed from the initial model. During this process, variables that did not significantly change the prevalence ratios and confidence intervals^9^ were removed until a final model was obtained, taking into consideration the p value (significance set at p < 0.05) and the 95% confidence interval (95%CI).

The following inclusion criteria were applied to select the sample: workers with complete data in the records on ML for own health treatment with clinical diagnosis specified according to the ICD-10 and number of days of absence recorded. Information on the workers harvested from the medical records was maintained in confidentiality and none of the workers are identified by name during the tabulation process. These data were then input to the database and coded numerically.

The data with secondary information were generated from the Federal Public Sector Workers’ Integrated Health Care Subsystem (Subsistema Integrado à Saúde do Servidor Público Federal - SIASS-Inconfidentes), Official Health Care Examination module, extracting only data on leave granted for treatment of own health to UFOP workers, by duration of absence (days), sex, causes of absence according to the ICD-10 (diagnosis), age group, and occupational category. The analysis is descriptive, using Excel spreadsheets (Microsoft Office 2019).

The research project was authorized by the Human Research Ethics Committee at UFOP with Ethics Appraisal Submission Certificate No. 19070619.1.00000.5150. None of the authors have any conflicts of interests related to the study.

## RESULTS

**Figure 1:**
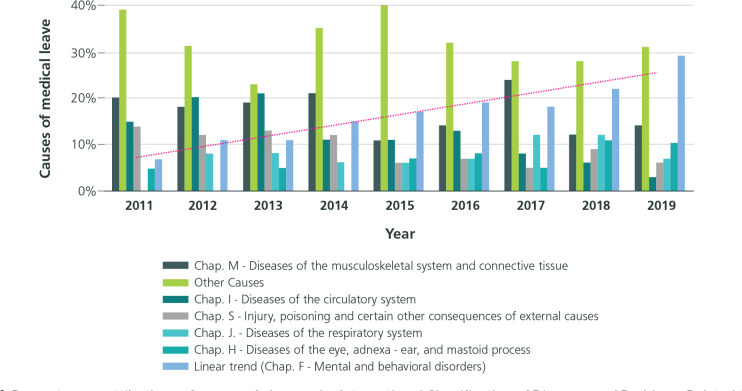
Percentage contributions of causes of absence by International Classification of Diseases and Problems Related to Health - 10th Revision (ICD-10) groups among employees of the Universidade Federal de Ouro Preto (UFOP), by the first four ICD-10 chapters ranked by magnitude, Ouro Preto, Brazil, 2011-2019. Source: SIAPENet Portal - Health module - Federal Public Sector Workers’ Integrated Health Care Subsystem - (Subsistema Integrado à Saúde do Servidor Público Federal - Inconfidentes SIASS)/UFOP.

The study sample comprised permanent members of the UFOP staff, which in 2019 totaled 972 faculty members and 763 ETAW, 58.7% of whom were male and 38.8% of whom were aged 31 to 40 years. The majority of the faculty members were male (60.2%) and the most prevalent age groups were 40 to 49 years among male faculty members and 30 to 39 years among female faculty members. The majority of the ETAW were male (57%) and the most prevalent age group was 30 to 39 years for both sexes. Distribution of the working hours of the educational technical and administrative workers revealed that 97% had a 40-hour working week. Other numbers of working hours are possible if the worker opts for a different administrative regime or in the case of professions that have a legally mandated number of weekly working hours (journalism, for example). It was found that UFOP has well-qualified technical and administrative staff, 80% of whom have a higher education qualification. Just 4.3% had not finished primary school, and 2.5% had only finished secondary school. A similar proportion was observed for the whole 9-year period analyzed. From 2011 to 2019, according to information from the Official Health Care Examination module of the FPSWIHS, the 791 workers with ML had a total of 3,805 absences for all ICD-10 causes, with a mean of 4.8 absences/worker. There was a total of 55,500 lost work days, with 12,420 days (22.8%) caused by absence for MBD. Female staff lost a total of 6,565 (52.9%) work days because of MBD and male staff lost 5,855 (47.1%) days. Distribution of absences from all causes listed in the ICD-10 according to the variables studied, reveled that the absences were more frequent among women (53.7%), when the Health Care Examination was conducted by a single physician (rather than a board) (77.5%), in workers aged 31 to 40 years (34.4%), and among faculty members (67.4%). The most common causes of absence by ICD-10 chapter in 2019 were MBD (29%); diseases of the musculoskeletal system (14%); cancers (10%); diseases of the eyes, adnexa, and ears (10%); and diseases of the respiratory apparatus (7%). During the 9-year period, the prevalence of absence due to MBD was approximately 20%. Figure 1 illustrates the historical series of percentages of absences for causes in the four most prevalent ICD-10 chapters.

The sample included in the initial collection of data from individual medical records that met the inclusion criteria comprised 730 workers, 23.2% (n = 170) of whom had ML for conditions classified in Chapter V of the ICD-10 - Group F (MBD). There were 491 ML events during the period caused by MBD. Majorities of the sample were females (57.6%) and ETAW (62.4%) and the most prevalent age group was 31 to 40 years (30%). With regard to the number of ML due to MBD, 61.2% of the sample had been absent more than once (Table 1). From 2011 to 2019, a total of 15,545 work days were lost due to ML because of MBD, with a mean of 91 days (SD = 138.2) and a median de 32 days. The workers had a mean of 3 absences for MBD, with a minimum of 1 absence, a maximum of 13 absences (SD = 2.7), and a median of 2. The mean time working at the institution before the first absence for MBD was 11.6 years (SD = 10.9) with a median of 7.2 years. The mean age at induction was 31.8 years. The most prevalent causes of absenteeism for MBD were depressive episodes and recurrent depressive disorder (F32 and F33) with 41.8%, followed by phobic-anxious disorders and other anxious disorders (F40 and F41) with 26.3%, and reactions to severe stress and adaptation disorders (F43) with 18.9%. Among the faculty members, the most prevalent causes were ICD codes F32 and F33 (43.4%), followed by ICD codes F40 and F41 (21.9%) and ICD code F43 (12.8%). Among grade “A” ETAW, the most important cause of absence for MBD was use of alcohol (F10), with 50%. Among grade “B”, “C”, “D”, and “E” workers, the most prevalent causes were depressive episodes and recurrent depressive disorder (F32 and F33), with 45, 29, 45, and 48% respectively. The most prevalent causes of absenteeism among male workers were ICD codes F40 and F41 (30.2%) and among female workers the most prevalent causes were ICD codes F32 and F33 (56.2%). No association was observed between the outcomes “number of absences for MBD” or “total number of days of absence due to MBD” and the explanatory variables sex, occupational category, age group, and time working at UFOP. Only the outcome “date of first absence for MBD” had a statistically significant association (p ≤ 0.05), with the variable time working at UFOP (Tables 2 and 3).

**Table 1:** Characteristics of workers at the Universidade Federal de Ouro Preto (n = 170) who took ML for mental and behavioral disorders, Ouro Preto, Minas Gerais, 2020

Characteristics	n	%
Sex		
Male	72	42.4
Female	98	57.6
Total	170	100.0
Occupational category		
Faculty members	65	38.2
ETAW grade A	4	2.3
ETAW grade B	11	6.5
ETAW grade C	21	12.3
ETAW grade D	39	23.0
ETAW grade E	30	17.6
Total	170	100.0
Faculty members by sex		
Male	25	39.1
Female	39	60.9
Total	64	100.0
ETAW by sex		
Male	49	46.2
Female	57	53.8
Total	106	100.0
Age groups (years)		
≤ 20	1	0.6
21-30	25	14.7
31-40	51	30.0
41-50	44	25.9
51-60	37	21.8
> 60	12	7.1
Total	170	100.0
Age groups, males (years)		
≤ 20	1	1.4
21-30	9	12.5
31-40	14	19.5
41-50	16	22.2
51-60	23	31.9
> 60	9	12.5
Total	72	100.0
Age groups, females (years)		
≤ 20	0	0.0
21-30	16	16.3
31-40	36	36.7
41-50	29	29.6
51-60	14	14.3
> 60	3	3.1
Total	98	100.0

ETAW = educational technical and administrative workers.

**Table 2:** Univariate analysis of the outcome “date of first absence for MBD”, with median values and prevalence ratios (with 95%CI) for occupational and sociodemographic variables, in workers at the Universidade Federal de Ouro Preto, Ouro Preto, Minas Gerais, Brazil, 2020

Variables	Values less than or equal to the median		Values greater than the median		Total		PR	95%CI	p-value
	n	%	n	%	n	%			
Sex (n = 167)									
Male	42	59.1	29	40.9	71	100.0	1.0		
Female	42	43.7	54	56.3	96	100.0	0.73	(0.52-1.01)	0.06
Occupational category (n = 167)									
Faculty member	31	50.0	31	50.0	62	100.0	1.0		
ETAW grade A	4	100.0	0	0.0	4	100.0	6.33^e-0.7^	(2.29^e-07^-1.74^e-06^)	0.00
ETAW grade B	8	72.7	3	27.3	11	100.0	0.54	(0.20-1.48)	0.23
ETAW grade C	10	47.6	11	52.4	21	100.0	1.05	(0.65-1.69)	0.85
ETAW grade D	17	43.6	22	56.4	39	100.0	1.13	(0.78-1.64)	0.53
ETAW grade E	14	46.7	16	53.3	30	100.0	1.07	(0.70-1.62)	0.76
Age group (n = 167) (years)									
≤ 39.38 years	8	14.8	46	85.2	54	100.0	1.0		
> 39.38 and ≤ 50.92	14	35.9	25	64.1	39	100.0	0.75	(0.58-0.98)	**0.03**
> 50.92	62	83.8	12	16.2	74	100.0	0.19	(0.11-0.32)	**0.00**
Time working at UFOP (years)									
≤ 11.35	14	16.7	70	83.3	84	100.0	1.0		
> 11.35	70	84.3	13	15.7	83	100.0	0.19	(0.11-0.31)	0.00

P-values marked with bold indicate that p < 0,20.

95%CI = 95% confidence interval; PR = prevalence ratio; ETAW = educational technical and administrative workers; MBD = mental and behavioral disorders; UFOP = Universidade Federal de Ouro Preto.

**Table 3:** Multivariate analysis of the outcome “date of first absence for MBD” - Poisson test, showing prevalence ratios, with 95%CI, by explanatory variables, “time working at UFOP”, in workers at the Universidade Federal de Ouro Preto, Ouro Preto, MG, Brazil, 2020

Tempriafastfcat^*^	PR	95%CI	p
Time working at the UFOP			
≤ 11.35 years	1.0		
> 11.35 years	0.19	0.11- 0.31	0.00

* Working time for the first categorized leave.

95%CI = 95% confidence interval; MBD = mental and behavioral disorders; PR = prevalence ratio; UFOP = Universidade Federal de Ouro Preto.

## DISCUSSION

The results of this study reveal a rising trend in absenteeism due to MBD in federal public sector workers at the UFOP, which is the number one cause of ML at the institution. Specific reasons for ML include depressive episodes (F32) and recurrent depressive disorder (F33). International studies show that depression and anxiety are the greatest causes of work absenteeism and are also responsible for higher health service expenditure when compared to other mental disorders.^10^ Possible causes of the increasing prevalence of absenteeism due to MBD include bullying, outsourcing combined with elimination of job titles considered obsolete in federal employees’ career paths, and the economic crises in Brazil and globally. Bullying is characterized by exposure of workers to humiliating and embarrassing situations, repetitively and for a long time, while performing their jobs. Such situations degrade workers’ dignity and psychological integrity. Sometimes these are minor aggressions which, if taken in isolation may be considered insignificant, but which are destructive when inflicted systematically.^11^ In 2008, the economic crisis in the United States spread to the rest of the world, and, in 2014, Brazil went through an economic and political crisis. In periods of economic crisis, factors that are protective of mental health are weakened, risk factors are strengthened, and mental health is impacted negatively. As socioeconomic status declines because of unemployment, impoverishment and debt, people experience increased uncertainty, insecurity, and loss, establishing despair and mental health problems. The subjectivity of the feelings of uncertainty (financial, occupational, and with relation to the future) and of loss (of income, of employment, of social networks, of socioeconomic status, and of self-esteem) is very often associated with access to health care that is objectively inadequate and late, culminating in establishment or exacerbation of a psychiatric disorder.^12^

One limitation of this research is linked to the type of study, which was epidemiological descriptive, and cannot identify temporal relationships between events. The fact that the study participants work in the tertiary sector of the economy and have employment stability means that the data cannot be extrapolated to the general population, although comparisons can be made with populations with similar characteristics. One strong point of this research is that the data used are from official records of absence from work because of health problems in federal public sector workers. Similar research conducted in the Southeast of Brazil and in other regions of the country confirms the magnitude of this problem, with high prevalence of MBD as cause of ML among public sector workers.^13^ With regard to the main causes of absenteeism by disease according to ICD-10 chapters, similar results were observed in other studies, such as in state level public sector workers from Santa Catarina state, from 1995 to 2005, among whom the most prevalent causes of absence according to the ICD-10 were MBD (25.3%), diseases of the musculoskeletal system and connective tissue (20.5%), and factors influencing health status and contact with health services (15.2%).^14^ In another study, the accumulated prevalence of ML by diagnostic group showed that MBD (26.5%), diseases of the musculoskeletal system and connective tissue (25.1%), and injury, poisoning and certain other consequences of external causes (23.6%) were the categories with greatest impact during the period.^15^ Although the order differs from the results of the present study, it will be observed that the groups are the same and that they differ from what is observed in the general population, among whom the number one cause of absence is injury, poisoning and certain other consequences of external causes (24%), including fractures, luxation, and traumatisms. The second-ranked cause is diseases of the musculoskeletal system and connective tissue (17%), and the third-ranked group is MBD (9%).^5^ In the present study, male workers primarily took ML for MBD when aged from 51 to 60 years, which is different from the overall result. Approximately two thirds of workers who took ML did so at least once more for MBD from 2011 to 2019. These data confirm findings in the literature. Prior episodes of ML increase the risk of subsequent absences for the same reason.^16^ In the present study, only the independent variable time working at the UFOP was associated with the outcome “date of first absence for MBD” in the final association model. In other words, UFOP workers who have been at the institution longer than the median (11.35 years) had a 19% protection factor compared to those who had been working at the UFOP for less than the median. In the literature, we found some studies that have investigated the association between absence from work for MBD and certain variables. A study with workers at a public university in provincial São Paulo state did not detect any association between age and absence due to MBD, but did find statistically significant associations between sex and absence due to MBD,^17^ and number of absences/year and absence due to MBD, with higher prevalence among participants with a greater number of absences.^17^ In another study, with workers from the Universidade Federal de Rondônia, in Porto Velho, an association was detected between the variables sex and age group and absence due to MBD.^18^ Work organization rating, introduction of new unknown technologies, workers’ expectations of the public institution, and a clear career path including role and responsibilities at the time of induction may have contributed to workers who had been at the UFOP for less time having a higher prevalence of ML for MBD. However, these hypotheses need investigation. Regarding the other variables (number of absences for MBD and total number of days of absence due to MBD) which did not exhibit associations in the final model, it is not possible to rule out increased disease severity, demanding more time for rehabilitation and recovery to return to work. As such, actions for promotion and prevention in mental health care should target all of the university’s permanent staff.

## CONCLUSIONS

The repercussions of ML because of problems affecting workers’ mental health have significant consequences and costs for workers, organizations, society, and the State (health care and social security systems), reducing productivity and increasing absenteeism and expenditure of treatment. It is important to implement preventative actions founded on interventions that target workers’ physical and mental health and actions related to providing support for woman. Such actions could include setting up a kindergarten and schools for their children, social integration with active leisure activities, activities for stress control, improvement of quality of life at work, changes to work organization, and creation of communication channels between the different levels of the hierarchy. The high prevalence of MBD detected in this investigation is an alert to the magnitude of the problem and constitutes evidence of the urgency of implementation of measures to detect psychosocial risk factors, whether associated with work or not. Finally, future investigations could seek explanations for the elevated percentage of absences due to MBD, which places this group of pathologies as the principal cause of absence among UFOP workers. Moreover, studies could also observe the relationship between MBD and the other frequent pathologies that affect federal public sector workers or the relation between MBD and other benefits granted by the government, in particular re-adaptation and removal.
